# Geographic Differences in the Dietary Quality of Food Purchases among Participants in the Nationally Representative Food Acquisition and Purchase Survey (FoodAPS)

**DOI:** 10.3390/nu11061233

**Published:** 2019-05-30

**Authors:** Maya Vadiveloo, Elie Perraud, Haley W. Parker, Filippa Juul, Niyati Parekh

**Affiliations:** 1 Department of Nutrition and Food Sciences, University of Rhode Island, Kingston, RI 02881, USA; haleyparker@uri.edu; 2 AgroParis Tech., 75231 Paris, Ile-de-France, France; elie.perraud@agroparistech.fr; 3 College of Global Public Health, New York University, New York, NY 10012, USA; fcj211@nyu.edu (F.J.); niyati.parekh@nyu.edu (N.P.); 4 New York University School of Medicine, New York, NY 10016, USA

**Keywords:** Food Acquisition and Purchase Survey (FoodAPS), diet quality, geographic disparities, racial/ethnic disparities, Healthy Eating Index, grocery purchase quality

## Abstract

Objective grocery transactions may reflect diet, but it is unclear whether the diet quality of grocery purchases mirrors geographic and racial/ethnic disparities in diet-related diseases. This cross-sectional analysis of 3961 households in the nationally representative Food Acquisition and Purchase Survey evaluated geographic and racial/ethnic disparities in grocery purchase quality. Respondents self-reported demographics and recorded purchases over 7 days; the Healthy Eating Index (HEI) 2015 assessed diet quality. Survey-weighted multivariable-adjusted regression determined whether there were geographic and racial/ethnic differences in HEI-15 scores. Respondents were, on average, 50.6 years, non-Hispanic white (NHW) (70.3%), female (70.2%), and had attended some college (57.8%). HEI-15 scores differed across geographic region (*p* < 0.05), with the highest scores in the West (57.0 *±* 0.8) and lowest scores in the South (53.1 *±* 0.8), and there was effect modification by race/ethnicity (*p*-interaction = 0.02). Regionally, there were diet disparities among NHW and non-Hispanic black (NHB) households; NHWs in the South had HEI-15 scores 3.2 points lower than NHWs in the West (*p* = 0.003). Southern NHB households had HEI-15 scores 8.1 points lower than Western NHB households (*p =* 0.013). Racial/ethnic disparities in total HEI-15 by region existed in the Midwest and South, where Hispanic households in the Midwest and South had significantly lower diet quality than NHW households. Heterogeneous disparities in the diet quality of grocery purchases by region and race/ethnicity necessitate tailored approaches to reduce diet-related disease.

## 1. Introduction

The Behavioral Risk Factor Surveillance System (BRFSS) has documented increasing geographic and racial/ethnic disparities in overweight, obesity, and type 2 diabetes prevalence [1], which contributes to cardiovascular morbidity and mortality in the US. Dietary risk factors, including low intake of plant-based foods and high intake of refined grains, added sugars, and animal products are the leading risk factors for these chronic diseases [2,3], making population-level interventions to improve diet paramount. Nevertheless, dietary patterns are influenced by multiple macro- and micro-system factors including the food environment, culture, and socioeconomic status. To date, most research on diet-related disparities has focused on micro-system-level factors like socioeconomic status rather than broader geographical characteristics, which may influence dietary behaviors through varying food environments and distinct regional foodways (i.e., learned food preferences, food access, and food demand) [4,5,6].

Recently, Kant et al. [7] evaluated secular trends in geographic differences in dietary intake in the National Health and Nutrition Examination Survey (NHANES). Consistent with observed disparities in chronic disease prevalence documented by the BRFSS, compared to the Northeast and West, respondents in the South and Midwest had higher intakes of energy-dense, nutrient-poor food groups and lower intake of fruits and vegetables. Moreover, other nutritional biomarkers also identified the Midwest and the South as having generally higher-risk dietary patterns in comparison to the West and Northeast—patterns that were consistent between 1988–1994 and 2009–2010. This evidence about the entrenchment of regional food patterns highlights a need to better understand regional foodways, identify region-specific healthy and unhealthy dietary behaviors, and develop region-specific interventions that can be monitored for effectiveness relatively seamlessly. Similarly, ingrained racial/ethnic disparities in diet quality [8,9,10] relatively resistant to intervention efforts necessitates greater contextualization of the drivers of food choice across high-risk groups, many of which are environmental [11]. This is particularly true given the variability observed across race/ethnicity in whether dietary adequacy and/or moderation recommendations are met [12], spurring further investigation about how differences in purchasing patterns and food preparation may contribute to observed differences [13].

A promising but understudied tool for monitoring diet quality is through the evaluation of food purchase data. Grocery purchases, while not perfectly correlated with intake [14], provide insight into the diet quality of home food environments, potentially providing a novel marker for understanding diet-related disease disparities at the population level [15]. The Food Acquisition and Purchase Survey (FoodAPS) [16] collected nationally representative food purchase data between 2012 and 2013 to better understand household food purchasing patterns and factors that influence such patterns, including census region and demographics (e.g., race/ethnicity), providing a unique opportunity to evaluate geographic disparities in diet. Given the powerful influence of regional foodways on dietary habits and rapidly evolving food environments, methodology able to measure regional differences in diet may be useful for monitoring and evaluating the influence of different interventions on dietary patterns in different regions of the US. Therefore, the purpose of the present study was to explore household-level differences in diet quality measured using the Healthy Eating Index 2015 (HEI-15) across the Northeast, South, Midwest, and West and to explore whether racial/ethnic differences in diet quality also exist across US geographic regions.

## 2. Materials and Methods 

### 2.1. Data Source

Nationally-representative grocery purchase data from the United States Department of Agriculture’s National Household Food Acquisition and Purchase Survey (FoodAPS) (2012–2013) were used [16]. The FoodAPS survey used a multistage sample design to recruit 14,317 individuals from 4826 households. Individuals who identified as the primary food shopper or meal planner were designated as the primary respondent (PR) for the survey, and responded to survey questions about household sociodemographic factors for all household residents and guests. The PR used hand scanners and/or food books to record all household food acquisitions during the 7-day survey period [16]. Data were evaluated by the FoodAPS research group, and for the 14.3% of purchased items where quantity was missing, data were imputed using information about food items, the stores from which they were purchased, and household characteristics [17]. For the present analyses, only purchasing events categorized as grocery purchases (i.e., foods acquired for at-home preparation and consumption) were evaluated. Additional details about FoodAPS design have been published previously [16,18]. 

### 2.2. Analytical Sample

The final analytic sample included 3961 households who provided data on grocery purchases. From the original sample (*n =* 4826), we excluded households who did not report data on grocery purchases (*n =* 459), households <10th percentile (*n =* 375) or >99.5th percentile (*n =* 24) of purchased items per week, households purchasing unidentifiable items (*n =* 6), and a single outlier whose total energy purchased exceeded the second greatest value by >15 times.

### 2.3. Healthy Eating Index 2015

The diet quality of grocery purchases was evaluated using the HEI-15 (range 0–100 points), with higher scores representing greater concordance with US Dietary Guidelines [19,20,21]. HEI-15 total and component scores for nine adequacy and four moderation components were computed using publicly available SAS code from National Cancer Institute [22]. Previous research suggests that a 5-point difference in HEI scores can be considered as clinically meaningful [11].

### 2.4. Exposures

The primary objective of this study was to evaluate whether the overall diet quality of household grocery purchases varied by geographic region and race/ethnicity in a manner similar to geographic disparities in obesity prevalence observed by region and race/ethnicity in the BRFSS. Therefore, we examined the associations between HEI-15 scores and census region (Northeast, Midwest, South, and West as defined by the Census 2010 boundaries [23]) and tested for effect modification between census region and the PR’s race (non-Hispanic white, non-Hispanic black, Hispanic, and Other/mixed race).

### 2.5. Covariates

Covariates were identified for possible inclusion in multivariable-adjusted models based on previous literature [24,25,26] and included variables at the PR- and household-level including age (PR), household size, household location (rural vs. urban), education level (PR), smoking (PR), participation in nutrition assistance programs, presence of overweight or obesity in the household, self-reported health status of household members, total energy purchased, and total number of items purchased.

### 2.6. Statistical Analysis

Descriptive statistics (means, standard deviations, and frequencies) were calculated for the sample. Survey-weighted linear regression using the FoodAPS strata, cluster, and survey sample weights as well as Taylor series linearization for variance estimation were used to calculate age-adjusted and multivariable-adjusted least square means and associated standard errors of the HEI-15 total and component scores. Covariates were each added individually to age-adjusted models and selected for inclusion in multivariable-adjusted models if the covariate notably changed estimated regional or racial/ethnic group means and R^2^. For every analysis, the coefficient of variation was calculated to ensure the reliability of survey estimates with values <0.3 considered reliable. Additionally, we tested for effect modification between census region and PR race/ethnicity using a threshold of *p =* 0.1 to determine significance. All analyses were conducted in SAS 9.4 (SAS Institute, Inc., Cary, NC, USA). A threshold of *p <* 0.05 was used to determine significance. For models with significant main effects, we conducted planned contrasts between meaningful subgroups.

## 3. Results

The sociodemographic characteristics of the analytical sample are presented in Table 1. On average, most household primary respondents identified as non-Hispanic white (70.3%), female (70.2%), had a mean age 50.6 years, had obtained a high school degree or attended some college (57.8%), and had a household income ≥130% of the federal poverty threshold (83.1%). There were differences in the demographic make-up of the household based on region of the country for race (*p <* 0.0001), food security status (*p =* 0.04), smoking (*p =* 0.02), rural vs. urban status (*p =* 0.02), and perceived healthfulness of the diet (*p =* 0.01). The proportion of primary respondents who identified as non-Hispanic white varied from 55.5% in the West to 82.3% in the Midwest. Descriptively, the proportion of households identifying as food secure was highest in the Northeast (90.4%), smoking prevalence was highest in the South (32.4%), rural locations were more frequent in the Midwest (42.0%), and the proportion of people who rated the healthfulness of their diet as excellent was highest in the West (14.3%).

**Figure 1:**
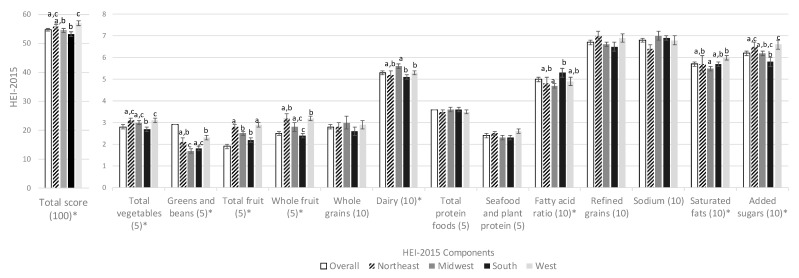
Unadjusted mean (SE) Healthy Eating Index 2015 component scores by region of food at home purchases of households participating in the National Household Food Acquisition and Purchase Survey 2012–2013, overall and according to census region (*N =* 3961). HEI-2015: Healthy Eating Index 2015; SE: standard error. Means and *p*-values calculated using unadjusted linear regression. * Indicates overall model significance (*p <* 0.05). Different superscripted letters indicate significant post-hoc differences (*p <* 0.05).

Unadjusted mean HEI-15 overall and component scores for all households according to geographic region are presented in Figure 1. The average HEI-15 total score in the overall population was 54.7 *±* 0.4 points. Compared to households in the South (53.1 *±* 0.8), households in all regions had higher diet quality of grocery purchases, but the difference was only statistically significant for households in the Northeast (55.7 *±* 0.8) and West (57.0 *±* 0.8). Households were furthest from meeting the targets for whole grains (2.8 out of 10 points), total vegetables (1.9 out of 5 points), and seafood and plant protein (2.4 out of 5 points), and closest to meet the targets for total protein foods (3.6 out of 5 points), sodium (6.8 out of 10 points), and refined grains (6.7 points). Regional differences in component scores were detected for all components except whole grains (*p =* 0.71), total protein foods (*p =* 0.86), seafood and plant proteins (*p =* 0.1), refined grains (*p =* 0.19), and sodium (*p =* 0.16).

**Figure 2:**
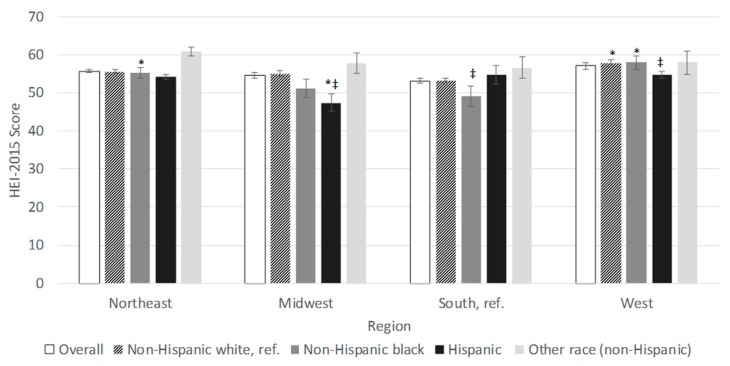
Unadjusted mean (SE) Healthy Eating Index 2015 total scores of food at home purchases by region of households participating in the National Household Food Acquisition and Purchase Survey 2012–2013, overall and according to census region (*N =* 3961). HEI-2015: Healthy Eating Index 2015; Ref.: reference group; SE: standard error. Means and *p*-values calculated using unadjusted linear regression. Missing values: * Indicates significantly different from the reference group (South). ^‡^Indicates significantly different from non-Hispanic white.

Regional differences in total HEI-15 scores were also detected by race/ethnicity (*p <* 0.0001) (Figure 2). Descriptively, NHW and NHB households had the highest HEI-15 scores in the West, while Hispanic households had the highest scores in the South and Other race households had the highest scores in the Northeast. In the Northeast, no racial/ethnic groups had significantly different HEI-15 scores from NHWs, whereas in the Midwest, Hispanic households had significantly lower scores than NHWs (47.4 *±* 2.4 vs. 55.0 *±* 0.7). In the South, NHB households had significantly lower scores than NHWs (49.1 *±* 1.7 vs. 53.2 *±* 1.9), and in the West, Hispanic households had significantly lower scores than NHW households (54.6 *±* 1.2 vs. 57.8 *±* 1.1). Using households in the South as the referent group, NHW households in the West had significantly higher HEI scores than NHW households in the South (57.8 *±* 1.1 vs. 53.2 *±* 0.9). The same pattern was observed for NHB households (57.9 *±* 3.0 in the West, 55.2 *±* 2.4 in the Northeast vs. 49.1 *±* 1.7 in the South, *p <* 0.05). However, for Hispanics, a pattern was reversed with households in the Midwest scoring significantly lower than households in the South (47.4 *±* 2.4 vs. 54.7 *±* 0.9).

**Figure 3:**
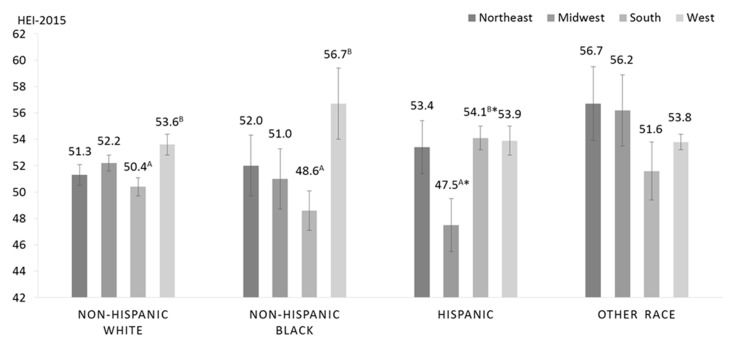
Multivariable-adjusted least square mean (SE) of total HEI-2015 score of household grocery purchases of households participating in the Food Acquisition and Purchase Survey (FoodAPS) (*N =* 3954), stratified by race/ethnicity and census region. Adjusted for age of the primary respondent, family income-to-poverty ratio, SNAP participation, any smoker in the household, education level of the primary respondent, and self-perceived health status of the primary respondent. Contrasts were used to conduct planned comparisons across different regions for each respective race/ethnicity group, using South as the reference region, as well as comparisons across racial groups in each region, using non-Hispanic white as the reference group. Different superscripted letters indicate significantly different from reference region (South) at *p <* 0.05. * Indicates significantly different from the reference group within region (non-Hispanic white) at *p <* 0.05.

Multivariable-adjusted least square mean HEI-15 total scores stratified by census region and race/ethnicity of the primary respondent are presented in Figure 3 (*p*-interactio*n =* 0.015). Contrasts were used to make planned comparisons across different regions for each respective race/ethnicity group using South as the comparison region, as well as comparisons across racial groups within each region, using NHW households as the reference group. 

Among NHW households, households in the South had HEI-15 total scores that were 3.2-points lower than households in the West (50.4 *±* 0.7 vs. 53.6 *±* 0.8 points, *p =* 0.003) after multivariable adjustment. Similarly, Southern NHB households had lower diet quality than NHB households in the West (48.6 *±* 1.5 vs. 56.7 *±* 2.7 points, *p =* 0.013) in multivariable-adjusted models. Southern NHB households had significantly lower diet quality than NHB households in the Northeast in the age-adjusted, but not the multivariable-adjusted, model (age-adjusted: 49.1 *±* 1.7 vs. 55.2 *±* 2.4, *p =* 0.047, multivariable-adjusted: 48.6 *±* 1.5 vs. 52.0 *±* 2.3, *p =* 0.23). Conversely, Hispanic households in the South had significantly higher diet quality than Hispanic households in the Midwest in both age-adjusted and multivariable-adjusted analyses (47.5 *±* 2.0 vs. 54.1 *±* 0.9 points, *p =* 0.005 in the multivariable-adjusted model). Diet quality did not differ significantly according to census region among households with primary respondents of Other race (Northeast: 56.7 *±* 2.8, Midwest: 56.2 *±* 2.7, South: 51.6 *±* 2.2, West: 53.8 *±* 0.6 in the fully adjusted model).

Compared to NHW households within the respective census region, NHB households in the South (53.2 *±* 0.9 vs. 49.1 *±* 1.7 points, *p =* 0.006) and Hispanic households in the West (57.8 *±* 1.1 vs. 54.6 *±* 1.2 points, *p =* 0.046) had significantly lower HEI-15 total scores in age-adjusted analyses. Nevertheless, the differences did not remain statistically significant when adjusting for smoking, SNAP participation, family income-to-poverty ratio (PIR) and age, education level and health status of the PR. Hispanic households in the Midwest and the South had significantly lower diet quality compared to NHW households in the respective census region (Midwest: 52.2 *±* 0.6 vs. 47.5 *±* 2.0 points, *p =* 0.021 South: 50.4 *±* 0.7, *p =* 0.007). Diet quality did not differ significantly according to race/ethnicity in the Northeast (NHW: 51.3 *±* 0.8, NHB: 52.0 *±* 2.3, Hispanic: 53.4 *±* 2.0, Other race: 56.7 *±* 2.8).

Table 2 presents overall and component scores by geographic region and race/ethnicity to better understand the contributions to disparities nationwide. Disparities in overall HEI-15 scores by race/ethnicity were not detected in the Northeast and West. Within the South, Hispanic households had higher overall HEI-15 scores (54.1 *±* 0.9) than NHW households (50.4 *±* 0.7, *p <* 0.05) whereas in the Midwest, Hispanic households had lower HEI-15 scores (45.5 *±* 2.0) than NHW households (52.2 *±* 0.6, *p <* 0.05). Within the South, Hispanic households had higher scores for total fruit (*p <* 0.001), whole fruit (*p <* 0.001), greens and beans (*p =* 0.002), sodium (*p =* 0.004), and saturated fat (*p =* 0.008), and lower scores for dairy (*p =* 0.013) than NHW households. Conversely, in the Midwest, Hispanic households had lower scores for total vegetables (*p =* 0.037) than NHW households.

Within each race/ethnicity, disparities were also detected across regions. For NHW and NHB households, overall HEI-15 scores were higher in the West compared to the South (*p <* 0.05). For NHW, higher scores in the West vs. South appeared to be driven by better total fruit (*p =* 0.004), whole fruit (*p =* 0.006), total vegetable (*p =* 0.04), and added sugar (*p =* 0.007) component scores whereas for NHB, higher scores in the West vs. South appeared to be driven by better total fruit (*p <* 0.001), whole fruit (*p <* 0.001), and refined grains (*p =* 0.03) scores. Among Hispanic households, HEI-15 scores were highest in the South (54.1 *±* 0.9) and significantly lower in the Midwest (47.5 *±* 2.0). Hispanic households in the Midwest vs. South had lower seafood and plant protein scores (*p =* 0.03) and marginally lower scores for total vegetables (*p =* 0.055) and total protein foods (*p =* 0.07).

## 4. Discussion

Analyzing purchasing data from the nationally representative FoodAPS survey provides a unique opportunity to explore geographic and racial/ethnic variation in the dietary quality of grocery purchases and inform the utility of monitoring grocery purchases to detect diet-related disparities nationally. In the present study, we detected clinically meaningful disparities in the diet quality of household food purchases according to geographic region and race/ethnicity. Notably, geographic and racial/ethnic disparities in diet quality were heterogeneous, underscoring the importance of accounting for such variation in the design and delivery of diet-related interventions. Although the South overall had the lowest HEI-15 scores, for Hispanic households, HEI-15 scores were highest in this region. Similarly, while NHW households generally had better HEI-15 scores than other races/ethnicities, Hispanic households had higher diet quality than NHW households in the South. Moreover, while NHB households generally had lower HEI-15 scores than NHW households, they had descriptively higher HEI-15 scores in the West, which did not differ from their NHW counterparts. There were no disparities apparent by race/ethnicity in the Northeast and the West, whereas in the Midwest, Hispanic households had HEI-15 scores more than 7.5 points lower than NHW households.

Differences in diet quality in the present study varied by region in a manner similar to what was published by the Global Burden of Disease Study [3], which generally found better health status and lifestyle behaviors among states in the West and Northeast than states in the South and Midwest. Similarly, data from the National Health Interview Survey found geographic variation in sugar-sweetened beverage intake, with the highest intake of carbonated sugary beverages in the South [27]. Sodas are the primary contributor to energy intake from sugary beverages according to data from NHANES, suggesting that energy intake from sugar-sweetened beverages may be highest in the South [28]. A recent study in NHANES also found that fruits, vegetables, and whole grain intake were higher in the West and Northeast than in the South and Midwest, which is consistent with higher overall diet quality [7]. 

The present study further investigated whether disparities in diet quality by region differed across racial/ethnic groups in the US. Interestingly, HEI-15 scores were highest in the South for Hispanic households; for NHW and NHB households, diet quality was highest in the West. In exploratory analyses examining differences in component scores by race/ethnicity and region, some patterns emerged that warrant further consideration. For NHB and NHW households in the West (vs. the South), differences were driven by moderation components and higher refined grains scores (among NHBs) and added sugar scores (among NHWs). In the South, differences in diet quality between Hispanic households and NHWs were primarily driven by adequacy components (fruits and vegetables), though Hispanic households also had lower sodium and saturated fat purchases. This finding somewhat aligns with data from the 2000–2012 Nielsen Homescan survey, which found that Hispanic and Black households in the US purchased less highly processed, ready-to-eat foods than their white counterparts and more foods for at-home preparation [13]. Taken together, the observed heterogeneity by geographic region and race/ethnicity both in overall and component HEI-15 scores components provide compelling evidence that dietary interventions may need to emphasize different aspects of the diet to improve the home food environment across varied groups in the US.

This relatively wide regional and racial/ethnic variation in diet quality likely reflects the divergent dietary patterns observed among NHB, Hispanic, and NHW populations in the US broadly [4,29,30]. Data from the Pew Research Center in 2014 indicate that the largest share of foreign-born Latinos live in the Southern states [31]. Given that factors such as country of origin, level of acculturation, and socioeconomic status influence dietary patterns, it is possible that Hispanic immigrants in the South have lower levels of acculturation compared to other regions, retaining more traditional dietary patterns associated with better overall diet quality, while Hispanic populations in the Midwest may be adopting more traditional US dietary patterns [32,33,34]. For NHB populations, foodways present in the “Stroke Belt and Buckle” may influence food choices among households and individuals differently than in other parts of the country [30]. While regional differences are less striking among NHWs, differences in diet quality between the South and West could be influenced by the role of Appalachian culture on the meaning of food for residents [4]. As a result, dietary interventions that use community-based approaches to develop tailored interventions reflective of sociocultural as well as regional patterns may be more successful [35].

Additionally, regional differences in the food environment and potentially marketing practices may also contribute to geographic and racial disparities [36,37]. For example, there is evidence that food environments with a higher density of fast food restaurants are associated with a higher prevalence of diabetes [38]. Furthermore, differences in public policy across the country may contribute to dietary norms in the region. For example, in regions where sugar-sweetened beverage taxes have been passed or are on the ballot (in the West and Northeast), this may influence social norms around beverages and reduce their purchase [39,40]. More research is needed to understand how factors in the built environment, which vary by region and potentially race/ethnicity, also influence the dietary quality of food purchases.

Some limitations of the present analysis must be noted. While the objective of this study was to evaluate a measure of the home food environment in a nationally representative sample, grocery purchases are moderately and imperfectly correlated with intake [14]. This study was cross sectional and exclusively focused on foods purchased for at-home consumption. Because the nutritional quality of restaurant meals is often less healthful than foods purchased for at-home consumption [39,41], it is possible that HEI-15 total and component scores are inflated. As such, the results presented likely underestimate observed geographic and racial/ethnic disparities—particularly among NHB adults and low- and middle-income households, where more frequent restaurant consumption has more adverse effects on total energy intake [42].

The present study also has numerous strengths. The FoodAPS survey is the first nationally representative sample of household grocery purchasing, and as a result, provides much-needed insight into disparities in food acquisitions at the household level across the US. To our knowledge, this is also the first study to explore differences in the diet quality of food purchases by geographic region. Data was rigorously collected over a 7-day period and linked with appropriate USDA food codes so that HEI-15 scores could be calculated. Because grocery purchase data is difficult to link with a nutrient database, the FoodAPS survey provides a unique opportunity to rigorously investigate the diet quality of grocery purchases. 

In conclusion, the dietary quality of grocery purchases varies in relation to race/ethnicity and geographic region in a nationally representative sample of US households. These disparities exist despite holding socioeconomic factors and education constant, underscoring the importance of addressing the higher levels of the social-ecologic model (i.e., macro-level environments (sectors), and settings) [43] when trying to modify determinants of food choice. The remarkable consistency of association between studies examining individual-level diet quality and household-level diet quality further highlight the promise of using grocery purchase data as an ongoing surveillance tool to monitor the diet quality of households. Moreover, the meaningful disparities in grocery purchase quality detected, as well as the overall low adherence to US Dietary Guidelines across all geographic and racial/ethnic groups, stress the need for ongoing monitoring and intervention to help combat the increasing burden of diet-related diseases nationwide [44]. 

## Figures and Tables

**Table 1:** Demographic, socioeconomic, and clinical characteristics of households participating in the National Household Food Acquisition and Purchase Survey 2012–2013 (*N =* 3961).

Characteristic	All Households	Northeast	Midwest	South	West	*p*-Value
	Mean ± SE	Mean ± SE	Mean ± SE	Mean ± SE	Mean ± SE	
***N***	3961	671	960	1427	903	
**Household size**	2.49 ± 0.05	2.5 ± 0.1	2.4 ± 0.1	2.4 ± 0.1	2.8 ± 0.2	0.1162
**Children (0–18 years) in HH**	0.64 ± 0.03	0.6 ± 0.1	0.6 ± 0.0	0.6 ± 0.1	0.7 ± 0.1	0.6713
**Race of primary respondent, %**						<0.0001
**Non-Hispanic white**	70.3	72.3	82.3	65.4	55.5	
**Non-Hispanic black**	9.9	8.19	9.38	13.3	5.84	
**Hispanic**	13.0	10.4	2.84	17.3	24.4	
**Other race (non-Hispanic)**	6.8	7.09	5.46	3.95	14.3	
**Sex of primary respondent, %**						0.0802
**Male**	29.8	24.1	31.0	30.8	31.1	
**Female**	70.2	75.9	69.0	69.2	68.9	
**Age primary respondent**	50.6 ± 0.53	51.9 ± 1.1	50.9 ± 1.1	50.1 ± 0.8	49.8 ± 1.1	0.4878
**Education level primary respondent, %**						0.0670
**Less than high school**	26.3	21.9	29.0	29.0	20.6	
**High school degree/some college**	33.2	26.3	34.6	34.2	34.7	
**Bachelor’s degree or higher**	40.5	51.8	36.4	36.9	44.7	
**Family income to poverty ratio, %**						0.1766
**<130 %**	16.9	12.2	16.2	20.5	15.7	
**130–349 %**	41.1	39.8	44.8	40.3	37.2	
**≥350 %**	42.0	48.0	39.0	39.2	47.1	
**SNAP participation, %**	12.7	10.4	12.3	14.4	12.3	0.3720
WIC participation,* %	27.0	24.1	24.1	28.4	30.6	0.7711
**Food security status, %**						0.0381
**Food secure household**	86.0	90.4	88.9	83.1	82.5	
**Food insecure household**	14.0	9.56	11.1	16.9	17.5	
**Smoker in HH, %**	29.3	24.4	31.4	32.4	24.0	0.0151
**Anyone obese in HH, %**	45.4	37.8	49.6	46.6	42.4	0.0737
**Self-perceived health status of primary respondent, %**						0.1226
**Excellent**	13.1	15.5	10.2	13.2	15.8	
**Very good**	34.5	37.4	35.9	32.4	33.6	
**Good**	36.0	34.3	40.2	35.6	30.9	
**Fair**	13.4	10.6	11.8	14.8	16.2	
**Poor**	3.02	2.27	1.95	4.10	3.48	
**HH located in rural census tract, %**	34.6	27.3	42.0	41.2	15.6	0.0205
**Total FAH purchases, kcal**	35615.9 ± 730.5	36623 ± 2853	34638 ± 1010	35108 ± 1094	37395 ± 2233	0.6792
**Total FAH items purchased**	33.1 ± 0.58	34.0 ± 1.3	32.7 ± 1.0	32.6 ± 1.1	33.7 ± 1.6	0.8172
**Perceived healthfulness of diet, %**						0.0089
**Excellent**	8.20	9.63	5.84	6.47	14.3	
**Very good**	29.6	33.2	28.9	28.2	30.5	
**Good**	42.0	40.6	45.1	42.4	37.2	
**Fair**	17.0	12.5	18.5	19.0	14.6	
**Poor**	3.11	4.05	1.60	3.88	3.42	

FAH: food at home; HH: household; SE: standard error; SNAP: Supplemental Nutrition Assistance Program; WIC: Special Supplemental Nutrition Program for Women, Infants, and Children. *Of WIC-eligible households (*n =* 896). All values are means *±* SE unless otherwise noted. The *p*-values were estimated by unadjusted linear regression, treating Healthy Eating Index (HEI) group as an ordinal variable, for continuous variables, and by Pearson’s chi-square for categorical variables. Missing values: race of primary respondent (*n =* 4), education of primary respondent (*n =* 3), SNAP participation (*n =* 1), anyone in HH receives benefits from WIC (*n =* 3100) smoking (*n =* 2), perceived healthfulness of diet (*n =* 2).

**Table 2:** Multivariable-adjusted mean (SE) Healthy Eating Index-2015 overall and component scores by census region and race/ethnicity of households participating in the National Household Food Acquisition and Purchase Survey 2012–2013 (*N =* 3654).

Region	South (reference)	Northeast	Midwest	West
**Total (100)**				
Non-Hispanic white (reference)	50.4 ± 0.7	51.3 ± 0.8	52.2 ± 0.6	53.6 ± 0.8 *
Non-Hispanic black	48.6 ± 1.5	52.0 ± 2.3	51.0 ± 2.3	56.7 ± 2.7 *
Hispanic	54.1 ± 0.9 δ	53.4 ± 2.0	47.5 ± 2.0 *δ	53.9 ± 1.1
**Adequacy components**				
**Total Fruit (5)**				
Non-Hispanic white (reference)	1.8 ± 0.1	2.2 ± 0.2*	2.2 ± 0.1 *	2.3 ± 0.2 *
Non-Hispanic black	1.8 ± 0.2	2.4 ± 0.2	1.7 ± 0.3	3.5 ± 0.3 *δ
Hispanic	2.4 ± 0.1 δ	2.5 ± 0.1	2.0 ± 0.3	2.8 ± 0.2
**Whole fruit (5)**				
Non-Hispanic white (reference)	2.0 ± 0.1	2.6 ± 0.2 *	2.5 ± 0.1 *	2.5 ± 0.2 *
Non-Hispanic black	1.6 ± 0.2	2.2 ± 0.2	2.0 ± 0.4	3.5 ± 0.3 *δ
Hispanic	2.8 ± 0.2 δ	2.7 ± 0.1	2.5 ± 0.5	3.1 ± 0.2
**Total vegetables (5)**				
Non-Hispanic white (reference)	2.6 ± 0.1	2.9 ± 0.1 *	2.9 ± 0.1 *	2.9 ± 0.1 *
Non-Hispanic black	2.7 ± 0.2	2.7 ± 0.2	2.7 ± 0.3	2.4 ± 0.3
Hispanic	2.9 ± 0.2	2.6 ± 0.3	2.2 ± 0.3 δ	3.0 ± 0.1
**Greens and beans (5)**				
Non-Hispanic white (reference)	1.4 ± 0.1	1.7 ± 0.2	1.4 ± 0.1	1.7 ± 0.1
Non-Hispanic black	1.4 ± 0.2	1.6 ± 0.3	1.3 ± 0.5	1.8 ± 0.5
Hispanic	2.0 ± 0.1 δ	2.1 ± 0.3	1.1 ± 0.5	2.1 ± 0.2 δ
**Whole grains (10)**				
Non-Hispanic white (reference)	2.4 ± 0.2	2.3 ± 0.2	2.5 ± 0.3	2.9 ± 0.3
Non-Hispanic black	1.8 ± 0.4	2.0 ± 0.6	2.5 ± 0.7	2.3 ± 0.5
Hispanic	2.1 ± 0.3	2.3 ± 0.2	2.4 ± 0.8	2.0 ± 0.2 δ
**Dairy (10)**				
Non-Hispanic white (reference)	5.6 ± 0.2	5.5 ± 0.3	6.0 ± 0.2	5.4 ± 0.2
Non-Hispanic black	3.7 ± 0.4 δ	3.3 ± 0.2 δ	4.1 ± 0.4 δ	5.0 ± 0.6
Hispanic	4.5 ± 0.4 δ	5.3 ± 0.3	5.6 ± 0.6	5.4 ± 0.3 *
**Total protein foods (5)**				
Non-Hispanic white (reference)	3.5 ± 0.1	3.4 ± 0.2	3.5 ± 0.1	3.4 ± 0.1
Non-Hispanic black	3.6 ± 0.2	3.3 ± 0.2	3.7 ± 0.2	3.7 ± 0.3
Hispanic	3.9 ± 0.2	3.8 ± 0.2	3.0 ± 0.5	3.6 ± 0.1
**Seafood and plant protein (5)**				
Non-Hispanic white (reference)	2.1 ± 0.1	2.2 ± 0.1	2.1 ± 0.1	2.3 ± 0.2
Non-Hispanic black	2.0 ± 0.3	2.3 ± 0.2	2.1 ± 0.2	2.7 ± 0.4
Hispanic	2.6 ± 0.2	2.4 ± 0.2	1.5 ± 0.4 *	2.2 ± 0.1
**Fatty acid ratio (10)**				
Non-Hispanic white (reference)	5.3 ± 0.2	4.8 ± 0.3	4.7 ± 0.2	4.9 ± 0.4
Non-Hispanic black	5.8 ± 0.4	6.1 ± 0.5 δ	5.9 ± 0.8	5.3 ± 0.7
Hispanic	5.6 ± 0.5	4.6 ± 0.3	4.1 ± 0.9	4.9 ± 0.3
**Moderation components**				
**Refined grains (10)**				
Non-Hispanic white (reference)	7.0 ± 0.3	6.2 ± 0.3 *	7.0 ± 0.2	7.0 ± 0.2
Non-Hispanic black	6.5 ± 0.5	6.4 ± 0.7	7.1 ± 0.4	7.9 ± 0.4 *δ
Hispanic	6.5 ± 0.3	5.7 ± 0.8	5.9 ± 0.6	5.8 ± 0.3 δ
**Sodium (10)**				
Non-Hispanic white (reference)	6.1 ± 0.2	6.3 ± 0.2	6.3 ± 0.2	6.4 ± 0.3
Non-Hispanic black	6.3 ± 0.5	7.9 ± 0.3 *δ	6.8 ± 0.3	7.1 ± 0.6
Hispanic	7.0 ± 0.4 δ	7.4 ± 0.7	6.7 ± 0.6	7.1 ± 0.4
**Added sugars (10)**				
Non-Hispanic white (reference)	5.6 ± 0.2	6.2 ± 0.3	2.2 ± 0.1	6.5 ± 0.3 *
Non-Hispanic black	5.3 ± 0.3	6.0 ± 0.3	5.8 ± 0.2	5.5 ± 0.7
Hispanic	6.0 ± 0.2	6.5 ± 0.4	5.3 ± 0.9	6.3 ± 0.4
**Saturated fats (10)**				
Non-Hispanic white (reference)	5.3 ± 0.2	5.2 ± 0.5	5.4 ± 0.2	5.6 ± 0.2
Non-Hispanic black	6.2 ± 0.3 δ	6.1 ± 0.3	5.5 ± 0.2	6.3 ± 0.5
Hispanic	5.9 ± 0.3 δ	5.9 ± 0.2	5.4 ± 0.5	5.8 ± 0.3

* denotes a significant difference (*p <* 0.05) in the mean *±* SE between the value of the HEI category from the South and another region for a given race. δ denotes a significant difference (*p <* 0.05) between the value of the HEI category from the non-Hispanic white and another race for a given region. Adjusted for age of the primary respondent, family income-to-poverty ratio, SNAP participation, any smoker in the household, education level of the primary respondent, and self-perceived health status of the primary respondent.
